# Effects of motivational music on sleep quality for caregivers of patients with advanced cancer: a randomized controlled trial

**DOI:** 10.1007/s00520-026-11038-6

**Published:** 2026-08-03

**Authors:** Inmaculada Valero-Cantero, María Ángeles Vázquez-Sánchez, Milagrosa Espinar-Toledo, Juan Corral-Pérez, José Luis Casals-Sánchez, Cristina Casals

**Affiliations:** 1 Nurse Case Manager, Puerta Blanca Clinical Management Unit, Malaga-Guadalhorce Health District, Malaga, Spain; 2 Department of Nursing, PASOS Research Group, Faculty of Health Sciences, UMA REDIAS Network of Law and Artificial Intelligence Applied to Health and Biotechnology, University of Malaga, Málaga, Spain; 3 Nurse, Limonar Clinical Management Unit, Malaga-Guadalhorce Health District, Malaga, Spain; 4 Present Address: ExPhy Research Group, Department of Physical Education, Instituto de Investigación E Innovación Biomédica de Cádiz (INiBICA), University of Cádiz, Cádiz, Spain; 5 Regional University Hospital of Málaga, Malaga, Spain; 6 Present Address: Instituto de Investigación Biosanitaria, ibs.GRANADA, Granada, Spain

**Keywords:** Accelerometry, Cancer, Caregivers, Palliative care, Self-reporting, Sleep

## Abstract

**Purpose:**

To investigate the benefits of listening to pre-bedtime music on sleep quality and daytime sleepiness for family caregivers of palliative care patients at home.

**Methods:**

Multicenter, randomized controlled clinical trial. Seventy-six caregivers were randomly assigned to intervention (*n* = 38) and control (*n* = 38) groups. The intervention group listened to individually chosen music for 30 min before bedtime for 7 days, and the control group listened to pre-recorded basic nursing education messages. The primary endpoint was the change in global subjective sleep quality, evaluated via the global Pittsburgh Sleep Quality Index (PSQI) score. Secondary endpoints included daytime sleepiness (Epworth Sleepiness Scale [ESS]), accelerometer-derived sleep parameters, and client satisfaction (CSQ-8).

**Results:**

Significant intergroup differences favoring the music intervention were observed in the mean changes from baseline for the primary endpoint of global sleep quality (PSQI Global Score: + 0.60 vs. + 2.05 points; *p* = 0.006). Descriptive intergroup differences favoring the intervention were observed for subjective nighttime sleep duration (+ 0.55 h; 95% CI 0.19 to 0.94) and daytime sleepiness (ESS: −2.74 points; 95% CI −4.11 to −0.56). Post-intervention satisfaction was significantly higher in the music group (CSQ-8: 27.61 vs. 24.63 points).

**Conclusions:**

Listening to pre-bedtime music maintains global subjective sleep quality for family caregivers in a home palliative care setting. Furthermore, exploratory signals suggest it may also prolong subjective sleep duration and reduce daytime sleepiness.

**Clinical trial registration:**

The study is registered at Clinical Trials.gov, NCT04491110. Date: 29 July 2020.

## Introduction

Family caregivers of patients with advanced cancer receiving palliative care may experience a range of challenges that disrupt their usual lifestyle and impact their overall health, such as burnout, exhaustion, anxiety [1], depression [2], diminished quality of life [3], or poor quality of sleep. Sleep quality refers to an individual’s subjective satisfaction with the restorative nature of their sleep, encompassing factors such as feeling refreshed upon waking, the duration of uninterrupted sleep, and the efficiency of sleep cycles throughout the night [4]. In addition, sleep quality plays a pivotal role in regulating various metabolic and physiological functions within the body [5].

Nonetheless, the quality of sleep may be prejudiced by social situations that provoke stress [6], problems with physical health [7], mental health disorders [8], or simply physiological aging [9]. For this reason, the prevalence of sleep-related problems is a significant concern, with nearly half of the population reporting such problems [10]. Caring for a family member with a terminal illness often leads to significant disruptions in sleep quality among caregivers, primarily due to emotional stress, physical demands, and the ongoing need for vigilance. Research indicates that family caregivers of terminally ill patients, especially those with cancer, frequently report poor sleep quality as they experience high levels of emotional distress and physical fatigue, both of which impact their ability to achieve restful sleep. For instance, caregivers' sleep quality worsened as the patient’s death approached, with increased rates of awakenings and shorter sleep durations due to caregiving responsibilities and emotional strain [11]. Another study with caregivers of cancer patients showed that 72.2% experienced poor sleep, citing emotional distress and financial worries as primary reasons for disturbed sleep [12].

Poor quality sleep can provoke many problems, including acute or chronic insomnia or insufficient total sleep time [13], and provoke many physical and mental alterations, such as heightening the risk of falls [14], aggravating the risk of gestational diabetes [15], provoking alterations to the lipid profile [16], or even giving rise to suicidal ideas [17]. Given these considerations, it is of vital importance to achieve and maintain good sleep quality.

Sleep disorders are most commonly addressed using pharmacological treatment, but this approach is only recommended as a short-term remedy, as it may provoke adverse side effects [18]. Accordingly, it is important to study the availability and effectiveness of non-pharmacological interventions, such as the use of music [19–21]. Various studies have evaluated the benefits of listening to music as a means of alleviating problems of poor sleep quality among students, adults, and the elderly [22–24]. However, the impact of music on specific populations, such as caregivers, remains less understood, particularly regarding variables like daytime sleepiness and accelerometer-measured parameters, including total sleep time, sleep efficiency, and sleep interruptions.

Therefore, this randomized controlled trial aimed to evaluate the efficacy of a passive music intervention among caregivers of patients receiving palliative care. The primary objective was to evaluate the impact of the intervention on the prespecified primary endpoint of global subjective sleep quality. The secondary objectives were to assess its supportive effects on secondary endpoints, including daytime sleepiness, accelerometer-measured sleep parameters recorded via actigraphy, and post-intervention satisfaction. The primary hypothesis of this trial was that family caregivers in the music intervention group, after listening to self-selected music before sleep for seven consecutive nights, would demonstrate a significant difference in global subjective sleep quality compared to the control group. Secondary hypotheses posited that, compared to the control condition, the music intervention group would experience a significantly greater reduction in daytime sleepiness, alongside favorable shifts across accelerometer-measured sleep architecture parameters captured via actigraphy.

## Methods

### Design

A multicenter, randomized, single-blind controlled clinical trial involving family caregivers of cancer patients in palliative care. The trial was registered prospectively on July 29, 2020, at ClinicalTrials.gov (Identifier: NCT04491110). The complete details of the prespecified trial methodology, endpoint outcomes, and operational protocols are publicly accessible in the previously published peer-reviewed study protocol [25].

### Participants

Participants in this study were family caregivers of advanced cancer patients receiving palliative home care through a healthcare program offered by six primary care clinics in the Province of Malaga. The sample was selected from the Palliative Care Assistance Process cancer patient lists. Caregivers meeting the inclusion criteria were contacted, informed about the study through verbal and printed explanations, and invited to participate.

Inclusion criteria: (1) family caregiver of a patient in in-home palliative care, (2) age ≥ 18 years. Exclusion criteria included (1) severe hearing loss impeding the use of headphones, (2) metal or plastic allergies, (3) planned absence during the 2-week study period, (4) lack of a mobile phone, and (5) status as a paid caregiver.

**Fig. 1:**
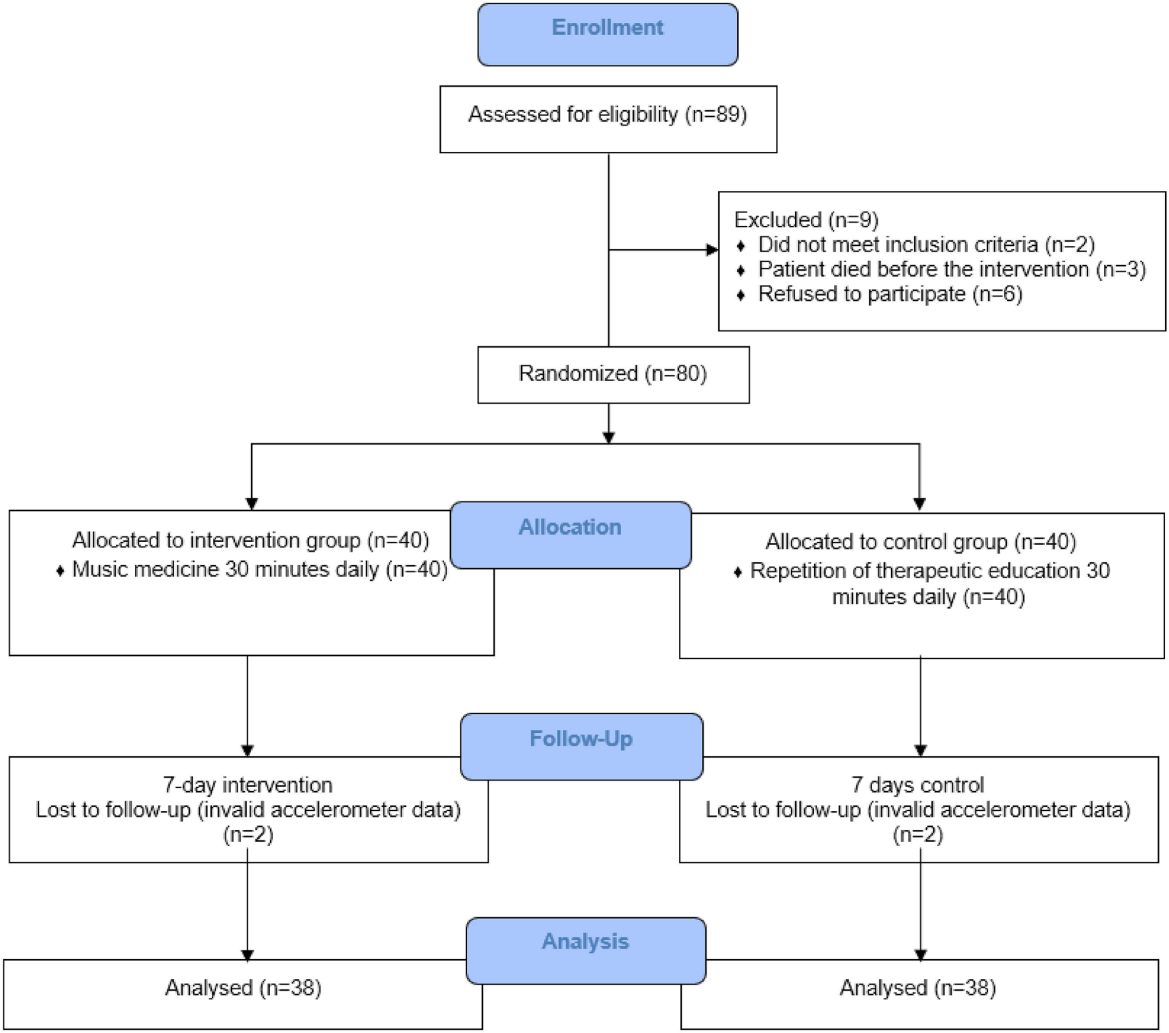
Flow chart for the study

The study flowchart is presented in Fig. 1.

### Ethics statement

The study adhered to the principles outlined in the Declaration of Helsinki [26] and received approval from the Research Ethics Committee of the Province of Malaga on May 15, 2018 (AP-0225-2019). All participants provided written informed consent prior to enrollment.

### Outcome measures

The primary endpoint of this trial was the change in global subjective sleep quality from baseline to post-intervention, measured using the validated global score of the Pittsburgh Sleep Quality Index (PSQI). Secondary endpoints included changes from baseline in daytime sleepiness, assessed via the Epworth Sleepiness Scale (ESS), and accelerometer-measured sleep metrics (time in bed, nighttime sleep duration, sleep efficiency, and sleep interruptions) captured continuously via actigraphy tracking. The final score of the 8-item Client Satisfaction Questionnaire (CSQ-8) served as an additional secondary endpoint evaluating intervention acceptability.

#### Primary outcomes

Perceived sleep quality was evaluated using the PSQI, specifically the validated Spanish-language version [27]. The PSQI consists of seven components that yield a total score ranging from 0 to 21, with higher scores indicating poorer sleep quality. A total score of 5 or less suggests optimal sleep quality, while a score of 6 or higher indicates sleep problems. Additionally, the assessment included variables such as sleep latency (the time taken to fall asleep), total nighttime sleep duration, time spent in bed, and sleep efficiency (the ratio of total sleep time to time spent in bed). Participants completed the PSQI at two time points, just before and just after the intervention, reporting their sleep patterns over the preceding 7 days. Both the control and intervention groups utilized the same questionnaire for consistency.

#### Secondary endpoints

##### Daytime sleepiness

Daytime sleepiness was assessed using the ESS, utilizing the validated Spanish-language version of the self-administered questionnaire [28]. This scale consists of eight questions, with responses scored from 0 to 3 points. Therefore, the total score can range from 0 to 24 points, with scores of 11 or higher indicating abnormal daytime sleepiness [29].

##### Sleep assessment by accelerometer-derived actigraphy

Accelerometer-derived sleep measurements were obtained using a triaxial accelerometer (Activinsights, GeneActiv) that participants wore continuously on their non-dominant wrist for a total of 14 days: 7 days before the intervention and another 7 days during the intervention. This methodology was applied consistently to both the control and intervention groups. The accelerometer was set to collect data at 40 Hz with a dynamic range of ± 8 g. An automated algorithm was utilized to detect the onset and offset of sleep periods by identifying sustained inactivity during the night [30]. The following variables were assessed: nighttime sleep duration (defined as the absence of a > 5° change in arm angle for 5 min or more), time in bed (the interval between sleep onset and wake-up time), sleep interruptions (measured as the number of awakenings and the duration of wakefulness lasting 5 min or longer after the onset of nocturnal sleep), and sleep efficiency [30].

##### Satisfaction with the intervention

Satisfaction with the attention received was measured using the Client Satisfaction Questionnaire (CSQ-8), employing the validated Spanish-language version [31]. This questionnaire consists of eight questions, each assessed on a scale from 1 to 4 points, resulting in a total score ranging from 8 to 32 points. While the total score is derived by summing the individual responses, it is important to note that four of the questions are reverse-scored.

### Sample size and randomization

Based on data from a systematic review examining the effects of listening to music on global subjective sleep quality [24], we estimated that a standard mean difference (SMD) of 0.776 for the global PSQI score, our primary endpoint, would be achieved. Assuming a two-sided independent two-sample t-test with a significance level (alpha error) of 0.05 and a statistical power (1-beta) of 0.90, we calculated that a sample size of 35 caregivers in each of the intervention and control groups (total *N* = 70) would be necessary to detect this effect (Epidat 3.1). In strict accordance with our prespecified, published protocol [25], to account for a potential dropout rate of approximately 15%, the final recruitment target was increased to 40 caregivers per group (*N* = 80).

To minimize bias in our analysis, participants were randomly assigned to either the experimental or control groups using a randomized mechanism. We prepared 80 cards, with 40 designated for the control group and 40 for the intervention group. These cards were placed in opaque envelopes, which were shuffled and numbered consecutively. Caregivers were assigned to the corresponding envelope in the order of their recruitment. This randomization procedure was conducted by the researcher responsible for the process, who explained the mechanism used but did not disclose whether the caregiver had been assigned to the control or intervention group.

### Procedures

Both the control and intervention groups received care according to the Palliative Care Plan published by the Andalusian Health Ministry [32]. This care included an initial assessment based on Virginia Henderson's 14 basic human needs, the development of a care plan to address any identified issues, and ongoing monitoring and evaluation. Additionally, each caregiver received training on fundamental aspects of care for both them and the patients they were supporting. This training encompassed health education in several areas, including nutrition and hydration, prevention and treatment of constipation, essential information about medications, measures to prevent pressure ulcers, general skin care, strategies to enhance sleep quality, recommendations for exercise and leisure activities, and techniques for effective communication.

#### Control group

The caregivers in the control group, in addition to receiving standard nursing care, were provided with access to audio files (hosted on Google Drive) that repeated essential educational content previously delivered by the nursing staff. A total of seven audio recordings, each lasting 30 min, were made available. Caregivers were instructed to listen to one recording each morning using headphones connected to their mobile phones.

#### Intervention group

The caregivers in the intervention group, in addition to receiving standard care, listened to music of their choice, guided by the criterion that the selected songs or tunes should evoke feelings of well-being. They were instructed to listen to this music for 30 min daily for 7 consecutive days, specifically just before going to sleep, and to refrain from engaging in any other activities during this time. The listening sessions were conducted using headphones connected to a mobile phone equipped with an app that allowed users to program the desired songs. Table 1 provides a characterization of the music chosen by each caregiver.

**Table 1:** Distribution of musical genres selected by the intervention group

Musical genre	*n* (%)
Flamenco	7 (18.4%)
Lounge	7 (18.4%)
Latin pop	6 (15.8%)
Romantic ballad	4 (10.5%)
Pop rock	3 (7.9%)
Celtic	2 (5.3%)
Classical	2 (5.3%)
Flamenco pop	2 (5.3%)
Opera	2 (5.3%)
US rock	1 (2.6%)
Spanish flamenco rock	1 (2.6%)
Traditional Hindu classical	1 (2.6%)

Data are presented as absolute frequencies and percentages, *n* (%). The total number of participants in the music intervention group analyzed was *n* = 38, of the total sample *N* = 76

#### Masking

Participants were not informed of their group assignment; however, those who listened to music might have inferred that they were in the intervention group, while those receiving health education messages may have deduced their status in the control group.

Evaluators were effectively blinded to the group assignments. All participants used identical headphones and mobile phones, with no apparent distinctions between the groups, and were instructed not to disclose to the evaluators what they had been listening to.

All evaluators were case management nurses from the participating primary healthcare units. The first evaluation occurred prior to group assignment, and the second evaluation took place immediately following the 7-day intervention period. Assessments were based on questionnaire responses and accelerometry results.

#### Data collection and analyses

Data for this study were collected from October 2020 to March 2022 during home visits. Prior to the intervention, subjective assessments of daytime sleepiness and sleep quality were conducted using the Epworth Sleepiness Scale and the PSQI, respectively. Accelerometer-measured outcomes were obtained through accelerometry-based actigraphy, performed continuously for 7 consecutive days.

Following the intervention, both daytime sleepiness and sleep quality were reassessed using the same methods, specifically for the 7-day duration of the intervention. Additionally, participants were asked to evaluate their satisfaction with the intervention using the CSQ-8.

Statistical analyses were performed using SPSS version 26.0 software. A per-protocol (PP) analysis approach was utilized, including only those participants who successfully completed their assigned 7-day protocol and provided complete baseline and post-intervention assessments.

A descriptive analysis of the study variables was conducted, presenting means and standard deviations for continuous variables, and frequencies and percentages for categorical variables. The normality of continuous variable distributions was assessed using the Shapiro-Wilk test.

Continuous clinical endpoints are presented as raw means and standard deviations. Individual change scores were computed directly at the intra-subject level by subtracting each participant's baseline value from their corresponding post-intervention value. Due to the mathematical properties of averaging individual changes within a sample, these values may differ slightly from the simple subtraction of the aggregated baseline mean from the aggregated post-intervention mean displayed in descriptive summaries.

To evaluate the primary endpoint (global PSQI score change), the Wilcoxon rank-sum test was utilized to compare the mean changes from baseline between the two independent study groups (intervention vs. control), with statistical significance set at *p* < 0.05. For all outcomes, treatment effects were quantified as the difference in mean change scores between the study arms. Given the non-normal distribution of individual change metrics, 95% confidence intervals (95% CI) for these treatment effects were constructed using a non-parametric percentile bootstrap method based on 5000 replications, providing a robust estimation independent of population normality assumptions (for the CSQ-8, CIs were derived using standard parametric assumptions).

Because the trial was explicitly powered only for the primary endpoint, formal hypothesis testing (*p*-values) was omitted for all secondary and exploratory outcomes. Consequently, these secondary endpoints were evaluated purely descriptively through their treatment effect estimates and corresponding 95% confidence intervals (CI).

## Results

The study results were obtained from 76 caregivers, divided equally between the intervention and control groups. Four of these participants (two from the control group and two from the intervention group) did not use the accelerometers properly and were excluded from the analysis (Fig. 1). None of the participants reported any adverse events. Analysis of the baseline data is shown in Table 2.

**Table 2:** Socio-demographic and baseline clinical characteristics of the caregivers

	Total (*N* = 76)	Intervention group (*n* = 38)	Control group (*n* = 38)
Categorical variables, *n* (%)			
Sex			
Female	66 (86.8%)	35 (92.1%)	31 (81.6%)
Male	10 (13.2%)	3 (7.9%)	7 (18.4%)
Education			
No formal education	11 (14.5%)	5 (13.2%)	6 (15.8%)
Primary	34 (44.7%)	18 (47.4%)	16 (42.1%)
Secondary	21 (27.6%)	10 (26.3%)	11 (28.9%)
Higher	10 (13.2%)	5 (13.2%)	5 (13.2%)
Employment status			
Employed	48 (63.16%)	23 (60.53%)	25 (65.79%)
Unemployed	13 (17.11%)	8 (21.05%)	5 (13.16%)
Retired	15 (19.74%)	7 (18.42%)	8 (21.05%)
Relation to the patient			
Spouse	45 (59.21%)	25 (65.79%)	20 (52.63%)
Son/Daughter	22 (28.95%)	8 (21.05%)	14 (36.84%)
Other	9 (11.84%)	5 (13.16%)	4 (10.52%)
Continuous variables, mean (SD)			
Age (years)	63 (13)	63 (13)	62 (12)
Daily care (hours)	17.29 (7.18)	18.03 (6.93)	16.54 (7.43)
Duration of palliative care (months)	3.87 (4.80)	4.71 (5.99)	3.03 (3.05)
PSQI			
Time in bed (hours)	7.84 (1.17)	7.70 (1.12)	8.00 (1.28)
Sleep latency (min)	23.02 (27.12)	27.46 (33.16)	18.58 (18.74)
Nighttime sleep (hours)	5.78 (1.60)	5.55 (1.61)	6.01 (1.57)
Sleep efficiency (%)	74.00 (19.00)	73.00 (19.00)	76.00 (20.00)
Sleep Quality (points)	9.05 (3.41)	9.37 (3.04)	8.74 (3.75)
ESS (points)	5.20 (4.354)	5.97 (4.46)	4.42 (4.16)
Accelerometer-derived sleep variables			
Time in bed (hours)	7.84 (1.08)	7.74 (0.93)	7.94 (1.21)
Nighttime sleep (hours)	6.66 (1.08)	6.63 (0.83)	6.68 (1.01)
Sleep efficiency (%)	85.20 (4.70)	86.10 (4.50)	84.30 (4.80)
Sleep interruptions (counts)	14.63 (3.43)	15.12 (3.79)	14.13 (3.00)
Total daily sleep (hours)	8.98 (1.63)	9.08 (1.62)	8.88 (1.66)

*ESS* Epworth Sleepiness Scale, *PSQI* Pittsburgh Sleep Quality Index, *SD* standard deviation

Regarding the primary endpoint, a statistically significant intergroup difference in the mean change from baseline favored the music intervention for global subjective sleep quality (PSQI Global Score: + 0.60 vs. + 2.05 points; *p* = 0.006) (Table 3).

**Table 3:** Intragroup variations and intergroup comparisons of clinical and sleep outcomes

	Intervention group (*n* = 38)			Control group (*n* = 38)			*p****-***value	Treatment effect (95% CI)
	Baseline	Post-intervention	Change (SD)	Baseline	Post-intervention	Change (SD)		
	Mean (SD)	Mean (SD)		Mean (SD)	Mean (SD)			
Primary outcomes								
PSQI								
Sleep Quality (points)	9.37 (3.04)	9.97 (3.48)	0.60 (1.97)	8.74 (3.75)	10.79 (3.86)	2.05 (2.09)	**0.006**	−**1.45 (**−**2.36; **−**0.56)**
Secondary outcomes								
PSQI								
Time in bed (hours)	7.70 (1.12)	7.76 (1.33)	0.06 (0.79)	8.00 (1.28)	7.86 (1.28)	−0.14 (0.61)		0.20 (−0.12; 0.53)
Sleep latency (min)	27.46 (33.16)	19.84 (19.75)	−5.61 (19.7)	18.58 (18.74)	17.82 (14.95)	−0.76 (14.3)		−4.84 (−12.97; 2.50)
Nighttime sleep (hours)	5.55 (1.61)	5.86 (1.36)	0.31 (0.97)	6.01 (1.57)	5.76 (1.60)	−0.24 (0.61)		0.55 (0.19; 0.94)
Sleep efficiency (%)	72.80 (18.80)	76.90 (18.10)	4.17 (11.9)	76.20 (19.90)	74.60 (20.10)	−1.62 (7.52)		5.79 (1.50; 10.42)
ESS (points)	5.97 (4.46)	4.29 (4.45)	−1.68 (2.47)	4.42 (4.16)	5.47 (4.94)	1.05 (3.15)		−2.74 (−4.11 −0.56)
Accelerometer-derived sleep variables								
Time in bed (hours)	7.74 (0.93)	7.77 (0.92)	0.03 (1.12)	7.94 (1.21)	7.75 (0.97)	−0.19 (1.04)		0.22 (−0.26;0.71)
Nighttime sleep (hours)	6.63 (0.83)	6.66 (0.78)	0.03 (0.99)	6.68 (1.01)	6.60 (0.74)	−0.08 (0.93)		0.11 (−0.32; 0.54)
Sleep efficiency (%)	86.10 (4.50)	86.40 (4.5)	0.30 (5.31)	84.30 (4.80)	86.12 (4.79)	1.80 (4.80)		−1.53 (−3.82; 0.67)
Sleep interruptions (counts)	15.12 (3.79)	14.33 (2.76)	−0.79 (2.83)	14.13 (3.00)	14.610 (2.38)	0.48 (2.71)		−1.27 (−2.50; −0.01
CSQ (points)		27.61 (3.32)			24.63 (4.11)			2.97 (1.27; 4.68)

*CI* confidence interval, *ESS* Epworth Sleepiness Scale, *PSQI* Pittsburgh Sleep Quality Index, *CSQ-8* 8-item Client Satisfaction Questionnaire, *SD* Standard Deviation. *p*-value derived from the Wilcoxon rank-sum test comparing the mean change scores (variation from baseline) between the intervention and control groups, except for CSQ, which is derived from an independent Student’s t-test comparing post-intervention scores between groups. Treatment effect values represent the difference in mean change scores between the study groups. The corresponding 95% CI for change parameters was constructed using a non-parametric percentile bootstrap method based on 5000 replications to robustly accommodate skewed distributions without assuming population normality, while the CSQ CI was derived using standard parametric independent t-test assumptions. Significant differences are highlighted in bold

For the secondary endpoints, among the specific subjective sleep components derived from PSQI, a descriptive intergroup difference was also observed for subjective nighttime sleep duration, which increased in the intervention group but decreased in the control group (treatment effect: + 0.55 h; 95% CI 0.19 to 0.94) and sleep efficiency (+ 5.79%; 95% CI 1.50 to 10.42). No other subjective PSQI component variations showed substantial descriptive differences. The analysis of daytime sleepiness yielded a intergroup difference in mean change scores, demonstrating a clear reduction in daytime drowsiness for the intervention arm compared with an increase in the control arm (−2.74 points; 95% CI −4.11 to −0.56). In terms of accelerometer-measured sleep metrics, a descriptive intergroup difference in change scores was observed for the number of nocturnal sleep interruptions, showing a reduction in the intervention group versus an increase in the control group (−1.27 counts; 95% CI −2.50 to −0.01). Other accelerometer-derived metrics did not show meaningful descriptive differences. Finally, post-intervention satisfaction scores were higher among participants in the music intervention group than among those in the control group (CSQ-8: + 2.97 points; 95% CI 1.27 to 4.68).

## Discussion

This study was undertaken to investigate the possible benefits of listening to music before going to sleep on the sleep quality and daytime sleepiness experienced by family caregivers for patients in in-home palliative care. Crucially, the primary endpoint of global subjective sleep quality, evaluated via the global PSQI score, demonstrated a statistically significant intergroup difference favoring the music intervention, driven by a stabilization of sleep profiles in the intervention group contrasted against a distinct deterioration of sleep quality in the control group. Secondary endpoints suggested that passive music intervention may improve subjective sleep time, reduce daytime sleepiness, and yield higher satisfaction with the treatment compared to the control group.

In the primary endpoint of the present study, overall sleep quality, as subjectively evaluated by the global PSQI score, a significant intergroup difference was found. The music intervention group maintained their overall sleep quality, while the control group experienced a distinct clinical decline, as indicated by an increased score on the questionnaire. This finding corroborates other investigations of the same question among different populations [33–35], although a recent meta-analysis of persons with no reported sleep disturbance found that, although the intervention was associated with improved sleep quality, the difference was not statistically significant [36].

Participants in the intervention group reported better descriptive subjective sleep efficiency, in line with previous work on this topic [35, 37]. Specifically, the intervention group reported a descriptive increase in the subjective duration of nocturnal sleep, demonstrating a mean change descriptively different from that observed in the control group. Consequently, this descriptive increase in nighttime sleep duration cannot be interpreted as definitive, confirmatory proof of efficacy. Instead, it should be viewed with appropriate caution as a supportive, exploratory signal that aligns with the positive direction of our primary outcome. Concerning sleep latency, although there was a slight decrease in the subjective evaluation of this parameter, the difference did not show a substantial descriptive effect, in contrast to other studies in this respect [35, 37]. However, our finding is consistent with another study in which sleep latency was measured objectively by polysomnography and found no significant differences following a pre-sleep audio intervention [38]. This indicates that for home-based palliative caregivers, the primary therapeutic value of pre-bedtime music lies in stabilizing sleep maintenance and mitigating daytime fatigue, rather than accelerating the immediate speed of sleep onset.

The descriptive assessments of daytime sleepiness among caregivers who listened to music before going to sleep indicated a clear improvement. However, this finding must be interpreted with appropriate clinical caution, because the caregivers’ baseline ESS scores were already within the normal, non-pathological threshold; this descriptive shift does not represent a treatment for clinical hypersomnia. Instead, it reflects a meaningful preventive stabilization of daytime alertness, which is an aspect of critical importance to the demanding performance of their caregiving roles. By mitigating the accumulation of daytime fatigue and preventing the deterioration of alertness observed in the control group, this simple intervention helps maintain caregiving vigilance, potentially reducing the risk of care-related errors and accidents to the mutual benefit of both the caregiver and the patient receiving home palliative care [22, 39, 40].

In contrast to subjective sleep outcomes, our actigraphy assessments did not show macro-structural improvements in total sleep efficiency or duration, a finding that conflicts with the PSQI results. This divergence highlights a well-documented variation between subjective perception and objective tracking in clinical sleep trials with caregivers [41]. However, a key finding was the descriptive intergroup difference in the change scores for nocturnal sleep interruptions. This outcome was not obtained in a similar study performed with actigraphy assessment, although the patients in this case suffered from insomnia [42]. However, caution is warranted when interpreting these accelerometer-measured sleep metrics. The descriptive reduction in nocturnal sleep interruptions in the intervention arm was small, averaging less than one awakening per night, and given that the trial was unpowered for this highly variable metric, it serves strictly as a descriptive observation. Nevertheless, the direction of these effects may still be clinically relevant as hypothesis-generating data. The reduction in nocturnal sleep interruptions, although averaging less than one awakening per night, suggests a potential improvement in sleep continuity among caregivers exposed to chronic caregiving-related stress [43]. This modest reduction in sleep fragmentation helps explain the parallel reduction in daytime sleepiness and the preservation of subjective sleep quality, even when total sleep duration remained unchanged. This divergence suggests that while the passive music intervention may not structurally alter macro-sleep architecture lengths, it deeply optimizes the qualitative perception of sleep depth and continuity. When caregivers experience fewer nocturnal awakenings, they frequently perceive their overall sleep tracking as more efficient and consolidated, even when automated accelerometer algorithms register stable overall durations. Future, larger-scale trials specifically powered for these individual dimensions are required to confirm whether these observed trends represent true, reproducible clinical improvements in sleep architecture.

Evaluating caregivers’ satisfaction with the attention received in this study is of fundamental importance to its application into clinical practice; if the intervention is positively valued by the caregivers, this suggests it would have beneficial consequences for their physical and/or mental situation [44]. In the present study, the participants who listened to music before going to sleep reported a descriptively higher degree of satisfaction with the attention received than those who listened to a repetition of basic health education messages on caregiving. This high satisfaction score underscores the high acceptability and non-invasive nature of music medicine, proving it to be a viable, patient-preferred digital health tool that can be easily integrated into the challenging daily routines of home-based palliative care providers.

This study is subject to some limitations. Firstly, the intervention lasted only 7 days, and a longer period might have produced a greater improvement in sleep quality. Secondly, we did not address whether the improvements observed would persist over time. A methodological nuance of this trial involves the temporal asymmetry between the study arms. While the intervention group listened to self-selected music immediately prior to bedtime, the control group listened to repeated nursing education recordings during the morning hours. This timing was intentionally chosen in the study design to prevent cognitive contamination at bedtime; it was reasoned that presenting educational medical information before sleep could increase the caregivers’ cognitive load or trigger care-related anxiety, thereby paradoxically disrupting their pre-sleep routine. Nevertheless, this temporal divergence means that the observed benefits in the music arm cannot be entirely decoupled from the behavioral effects of establishing a dedicated, calming bedtime routine itself. Future trials should explore alternative active control conditions that offer non-cognitive bedtime relaxation to further isolate the specific auditory mechanisms of music from general behavioral wind-down effects. Nevertheless, the clinical trial can be replicated straightforwardly. Moreover, the study conditions were rigorous, with the evaluator blinded to the intervention/control group assignments and caregivers not informed of their group assignments.

In summary, our findings demonstrate that listening to pre-recorded music before going to sleep maintains global subjective sleep quality. Furthermore, exploratory signals suggest it may also prolong subjective sleep duration and reduce daytime sleepiness according to subjective assessments. Further study is warranted to elucidate the factors underlying these differences between subjective perceptions and accelerometer-measured assessments of sleep architecture in high-stress caregiving cohorts. A notable finding of our study is that caregivers reported exceptionally high levels of satisfaction with the musical intervention, coupled with an excellent safety profile, as no adverse effects were recorded.

## Data Availability

The data that support the findings of this study are available from the corresponding author upon reasonable request.
